# Vanillin production using metabolically engineered *Escherichia coli *under non-growing conditions

**DOI:** 10.1186/1475-2859-6-13

**Published:** 2007-04-16

**Authors:** Paolo Barghini, Diana Di Gioia, Fabio Fava, Maurizio Ruzzi

**Affiliations:** 1 Department of Agrobiology and Agrochemistry, University of Tuscia, via Camillo de Lellis – snc, 01100 Viterbo, Italy; 2 DICASM, Faculty of Engineering, University of Bologna, Bologna, Italy

## Abstract

**Background:**

Vanillin is one of the most important aromatic flavour compounds used in the food and cosmetic industries. Natural vanillin is extracted from vanilla beans and is relatively expensive. Moreover, the consumer demand for natural vanillin highly exceeds the amount of vanillin extracted by plant sources. This has led to the investigation of other routes to obtain this flavour such as the biotechnological production from ferulic acid. Studies concerning the use of engineered recombinant *Escherichia coli *cells as biocatalysts for vanillin production are described in the literature, but yield optimization and biotransformation conditions have not been investigated in details.

**Results:**

Effect of plasmid copy number in metabolic engineering of *E. coli *for the synthesis of vanillin has been evaluated by the use of genes encoding feruloyl-CoA synthetase and feruloyl hydratase/aldolase from *Pseudomonas fluorescens *BF13. The higher vanillin production yield was obtained using resting cells of *E. coli *strain JM109 harbouring a low-copy number vector and a promoter exhibiting a low activity to drive the expression of the catabolic genes. Optimization of the bioconversion of ferulic acid to vanillin was accomplished by a response surface methodology. The experimental conditions that allowed us to obtain high values for response functions were 3.3 mM ferulic acid and 4.5 g/L of biomass, with a yield of 70.6% and specific productivity of 5.9 μmoles/g × min after 3 hours of incubation. The final concentration of vanillin in the medium was increased up to 3.5 mM after a 6-hour incubation by sequential spiking of 1.1 mM ferulic acid. The resting cells could be reused up to four times maintaining the production yield levels over 50%, thus increasing three times the vanillin obtained per gram of biomass.

**Conclusion:**

Ferulic acid can be efficiently converted to vanillin, without accumulation of undesirable vanillin reduction/oxidation products, using *E. coli *JM109 cells expressing genes from the ferulic acid-degrader *Pseudomonas fluorescens *BF13. Optimization of culture conditions and bioconversion parameters, together with the reuse of the biomass, leaded to a final production of 2.52 g of vanillin per liter of culture, which is the highest found in the literature for recombinant strains and the highest achieved so far applying such strains under resting cells conditions.

## Background

Flavours and fragrances are frequently used in the food, feed, cosmetic, chemical and pharmaceutical industries. Many flavour compounds are produced by chemical synthesis or by extraction from plant and animal sources. The major drawback of chemical synthesis is that the process is not environmentally friendly and that the compounds of interest often occur as undesirable racemic mixtures [1]. On the other hand, bioflavours are often present in animal and plants at low concentrations, making isolation and purification very expensive. Other bio-routes for flavour synthesis are based on microbial fermentation processes or on bioconversions of natural precursors using tailored microbial cells or enzymes [2]. In particular, microbial biocatalysis can be used for the production of many flavouring and fragrance aromatic compounds such as vanillin, benzaldehyde, lactones and methyl-ketones [1,3].

Vanillin (4-hydroxy-3-methoxybenzaldehyde) is the major organoleptic component of vanilla flavour which is extracted from the cured beans of *Vanilla planifolia*. It is one of the most important flavour compounds, and the current market demand is supplied mostly using synthetic vanillin, chemically produced from guaiacol and lignin [4], while natural vanillin obtained from *Vanilla *represent less than 1% of the annual market demand. Moreover, consumer desire for healthy and natural products and the fact that plant derived vanillin is relatively expensive, has led to the investigation of other biotechnological routes such as the microbial production of this flavour from phenolic stilbenes, lignin, eugenol and ferulic acid [5].

The latter, a cinnamic acid derivative, is a major component of lignin in plants and is also extremely abundant in the cell walls of many cereals and grasses [6]. Several microorganisms have been reported to transform ferulic acid to vanillin but, in many cases, vanillin is rapidly converted to less toxic products such as vanillic acid or vanillyl alcohol. Aldehydes, in general, are rarely accumulated in biological system because of their rather high chemical reactivity. The highest vanillin production from ferulic acid (more than 10 g/L with a molar yield of about 75%) was obtained with actinomycetes, such us *Amycolatopsis *sp. HR167 [7] and *Streptomyces setonii *ATCC 39116 [8]. However, filamentous growth of actinomycetes, resulting in highly viscous broths, unfavorable pellet formation and uncontrolled fragmentation and lysis of the mycelium, might complicate the rheology of the production processes, reduce their productivity and determine an increase in the downstream processing costs [9]. Unicellular microorganisms, such as *Pseudomonas*, do not show the same cultivation and scaling up problems, but have in general a lower productivity with respect to actinomycetes, as they tend to further transform vanillin to vanillic acid [10]. Attempts to prevent oxidation of vanillin by inhibition of vanillin dehydrogenase by dithiothreitol were of limited success [11].

The increasing knowledge regarding enzymes that are responsible for the conversion of ferulic acid to vanillin, as well as the identification and characterization of the genes coding for them [10,12,13], offers new opportunities for metabolic engineering and for the construction of recombinant strains carrying the genes encoding for the bioconversion of ferulic acid to vanillin, thus avoiding vanillin further oxidation. Only a few examples of vanillin production from genetically engineered strains have been described in the literature so far, and some of them show a low vanillin productivity [14] or reduction of vanillin to vanillyl alcohol [15]. More recently, Overhage et al. [16] proposed a two step process catalyzed by two recombinant *E. coli *strains for the production of vanillin from eugenol: in the first step ferulic acid was obtained from eugenol by *E. coli *XL1-Blue (pSK*vao*mP*calA*m*calB*) with a high molar yield (93.3%), while in the second step a second strain, *E. coli *(pSK*ech*E/H*fcs*), converted ferulic acid to vanillin. However, the yield of the second process was low and, in addition, the product was completely reduced to vanillyl alcohol. In 2005, Yoon et al. [17] have developed two recombinant *E. coli *strains by inserting the *fcs *(feruloyl-CoA synthetase) and *ech *(enoyl-CoA hydratase/aldolase) genes from *Amycolatopsis *sp. strain HR104 and *Delftia acidovorans *under the control of the arabinose-inducible promoter P_BAD _into the pBAD24 expression vector. The highest vanillin production was obtained with the *E. coli *strain carrying the *Amycolatopsis *genes, thus obtaining, under optimized growing-cell conditions, 580 mg/L of vanillin from 1 g/L ferulic acid. These studies indicate that recombinant strains could represent an interesting alternative to natural strains in the field of vanillin production. However, further studies are necessary in this field to develop other new strains able to provide higher productivity, strain stability and process selectivity.

In this work vanillin production obtained with resting cells of recombinant *E. coli *strains transformed with either a low copy and a multicopy vector containing the *ech *and *fcs *genes from *P. fluorescens *BF13 [18], together with their own promoter, was compared. Some parameters influencing the bioconversion process were optimized by employing the *E. coli *strain showing higher productivity. Finally, the response surface methodology was used to optimize vanillin yield and biomass productivity by selecting biomass and substrate concentration as variables.

## Results

### Determination of plasmid copy number

**Figure 1:**
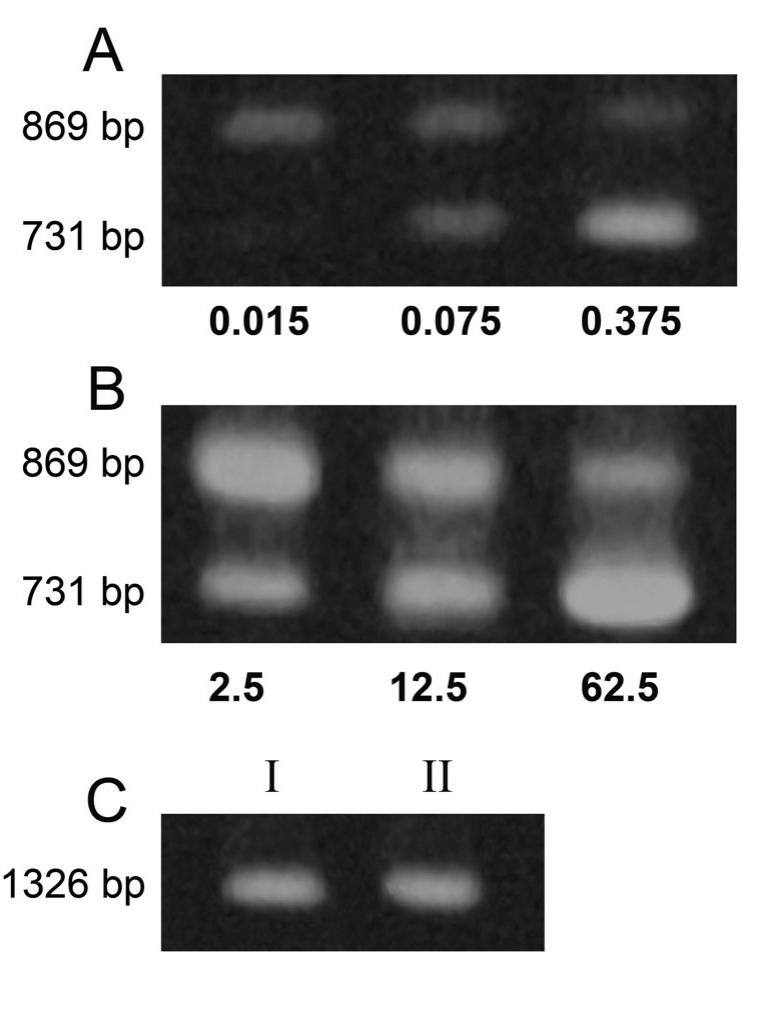
**Determination of plasmid copy number by competitive PCR**. Competitive PCR using total DNA from JM109 cells harbouring pBB1 (A) or pFS (B) plasmid with the indicated amount (ng) of competitor DNA. (C) PCR reactions with primers targeting to 16S rRNA (1326-bp band): I, JM109(pBB1); II, JM109(pFS). The amplification products were separated by electrophoresis on a 1.0% agarose gel and visualized after staining with ethidium bromide. The 869-bp band corresponds to the *ech *gene and the 731-bp band to its 138-bp deletion form.

Competitive PCR experiments were performed to approximate the number of copies of the exogenous competitor required to amplify equivalent amount of *ech *gene using total DNA from cells of *E. coli *JM109(pFS) and JM109(pBB1). The experiments (Figure 1, panels A and B) indicated that, when the competitive PCR was performed with a fixed amount of total DNA [2.5 × 10^7 ^cell equivalents], equivalent signals for the target and the competitor DNA were obtained with markedly different amounts of competitor: 9.5 × 10^7 ^copies (0.075 ng), with DNA from strain JM109(pBB1), and 1.58 × 10^10 ^copies (12.5 ng), with DNA from strain JM109(pFS), respectively. Amplification of 16S rDNA fragments yielded bands of the same intensity, thus confirming that an equivalent number of genomes was present in both DNA samples (Figure 1C). These results indicate that the copy number of pBB1 in JM109 cells is about four copies per genome and that the ferulic catabolic genes were present at 166-fold higher copy number in cells harbouring plasmid pFS.

### Bioconversion activity of recombinant E. coli

**Figure 2:**
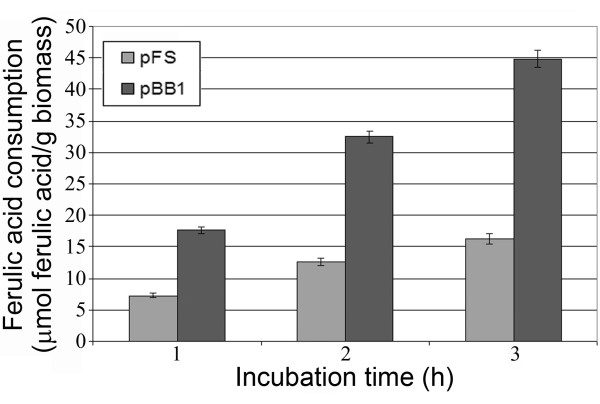
**Effect of host/vector system on the depletion of ferulic acid**. Experiments were carried out with 4 g (wet weight)/L biomass grown to stationary phase in LB medium. Resting cells of JM109(pFS) and JM109(pBB1) were incubated for 3 h in the presence of 0.5 mM ferulic acid and the depletion of the substrate was determined by HPLC analysis.

A 4 g(wet weight)/L cell suspension of both *E. coli *JM109(pFS) and JM109(pBB1) was used for vanillin production in the presence of 0.5 mM ferulic acid. Substrate consumption curves by resting cells indicated (Figure 2) that JM109(pBB1) consumed ferulic acid 2- to 3-fold more efficiently than *E. coli *strain expressing ferulic catabolic genes from a high-copy plasmid (pFS). The production of vanillin was maximum after 3 hours of incubation and was higher using JM109(pBB1) (molar yield of 29.6%) than JM109(pFS) cells (molar yield of 8.8%). Therefore, JM109(pBB1) was used as biocatalyst in the successive experiments.

### Optimization of vanillin production in shaken flasks

**Figure 3:**
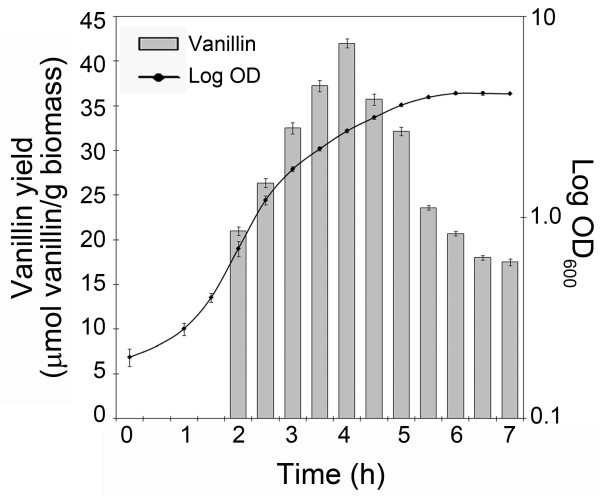
**Dependence of vanillin production on the stage of cell growth**. Bioconversion experiments were carried out using JM109(pBB1) cells [4 g(wet weight)/L] at different stage of growth. Resting cells were incubated for 1 h in the presence of 0.5 mM ferulic acid and the production of vanillin was determined by HPLC analysis.

**Figure 4:**
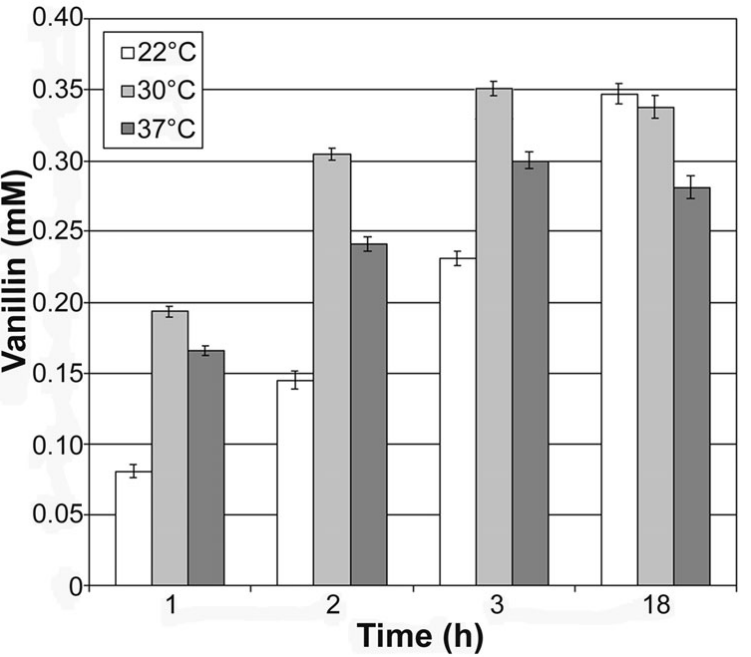
**Effect of the incubation temperature on the vanillin production by resting cells of JM109(pBB1) strain**. Bioconversion experiment were carried out using cells [4 g (wet weight)/L] from exponential-phase culture (OD_600 _= 2.7) which were incubated in the presence of 0.5 mM ferulic acid at different temperature.

The influence of the physiological state of the cells employed on the bioconversion yield in *E. coli *JM109(pBB1) strain was investigated in shaken flasks. For this purpose, bioconversion assays were performed with cells collected from cultures grown at different optical densities (Figure 3). Vanillin was quantified after 1 hour incubation in the presence of ferulic acid. The results (Figure 3) showed that the highest bioconversion yield was obtained with cells grown at an optical density at 600 nm of 2.7 (during growth transition to stationary phase). A 2-fold decrease in vanillin formation was observed when cells were harvested at higher (OD_600 _of 4.15; stationary phase) or lower (OD_600 _of 0.7; early exponential growth phase) optical densities (Figure 3). The temperature of the bioconversion assay was found to markedly influence vanillin production yield. Among the three temperature tested (37°C, 30°C and 22°C), the highest vanillin production was achieved at 30°C with a molar yield of 70.4% after 3 hour of incubation (Figure 4).

### Optimization of vanillin production using response surface methodology (RSM)

The bioconversion process was further optimized by studying the effect of biomass and substrate concentration employing the response surface methodology. The two studied parameters were entered into the design in Modde 5, which generated a full-factorial screening design with a total of 12 experiments including three replicate centre points. Molar yield and specific productivity were the two responses studied using central composite design (CCD). Range of variation of biomass was set from 1 to 8 g (wet weight)/L and the substrate concentration varied from 0.5 to 5 mM as described in Table 1. Cells grown in a 2-L bioreactor at 30°C were employed in the study. The growth temperature was lowered to 30°C as higher bioconversion rates and yields were observed with cells grown at this temperature with respect to 37°C (data not shown), although, in this condition, the rate of growth was lower and allowed to reach the desired physiological state (corresponding to an OD_600 _of 2.7) after 5 hours instead of 4. Bioconversion assays were performed in the experimental conditions previously optimized.

**Table 1:** Experimental design of RSM studies using two independent variables with three centre points, showing observed and predicted values of vanillin molar yield and specific productivity.

			Vanillin Molar yield (%)		Specific Productivity (μmol vanillin/g biomass × min)	
Experiment	Biomass (g/L)	Ferulic acid (mM)	Observed	Predicted	Observed	Predicted
1	1.0 (-1)	0.5 (-1)	65.4	66.4	4.27	4.01
2	4.5 (0)	0.5 (-1)	75.3	74.9	1.61	1.90
3	8.0 (1)	0.5 (-1)	68.0	66.9	0.88	0.67
4	1.0 (-1)	2.8 (0)	54.4	52.6	6.92	7.33
5	4.5 (0)	2.8 (0)	68.4	68.3	5.94	5.80
6	8.0 (1)	2.8 (0)	65.0	67.4	4.57	4.79
7	1.0 (-1)	5.0 (1)	33.1	33.7	5.81	5.73
8	4.5 (0)	5.0 (1)	55.7	56.5	4.58	4.60
9	8.0 (1)	5.0 (1)	63.9	62.6	4.10	4.08
10	4.5 (0)	2.8 (0)	68.4	68.3	5.94	5.80
11	4.5 (0)	2.8 (0)	68.5	68.3	5.94	5.80
12	4.5 (0)	2.8 (0)	68.2	68.3	5.94	5.80

(-1), (0) and (1) are coded levels

It was observed that the experimental and predicted values for both the responses were similar (Table 1). The ANOVA test for molar yield (*R*^2 ^of 0.989 and *Q*^2 ^of 0.898) and specific productivity (*R*^2 ^of 0.977 and *Q*^2 ^of 0.801) indicated that the model was significant.

**Figure 5:**
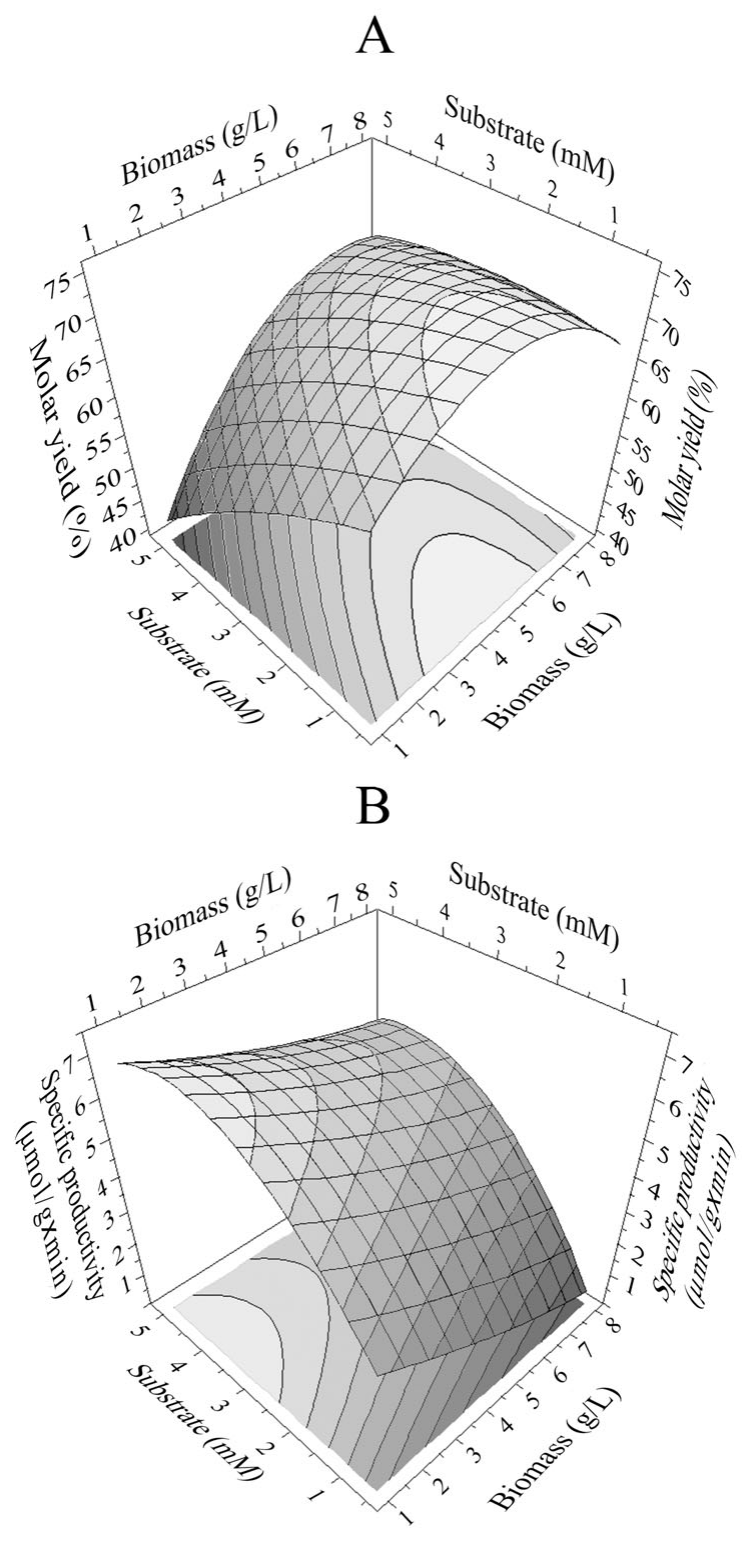
Mutual effect of biomass and substrate concentration on the molar yield (A) and the specific productivity (B) of vanillin evaluated as surface response calculated by the use of Modde 5.0.

**Figure 6:**
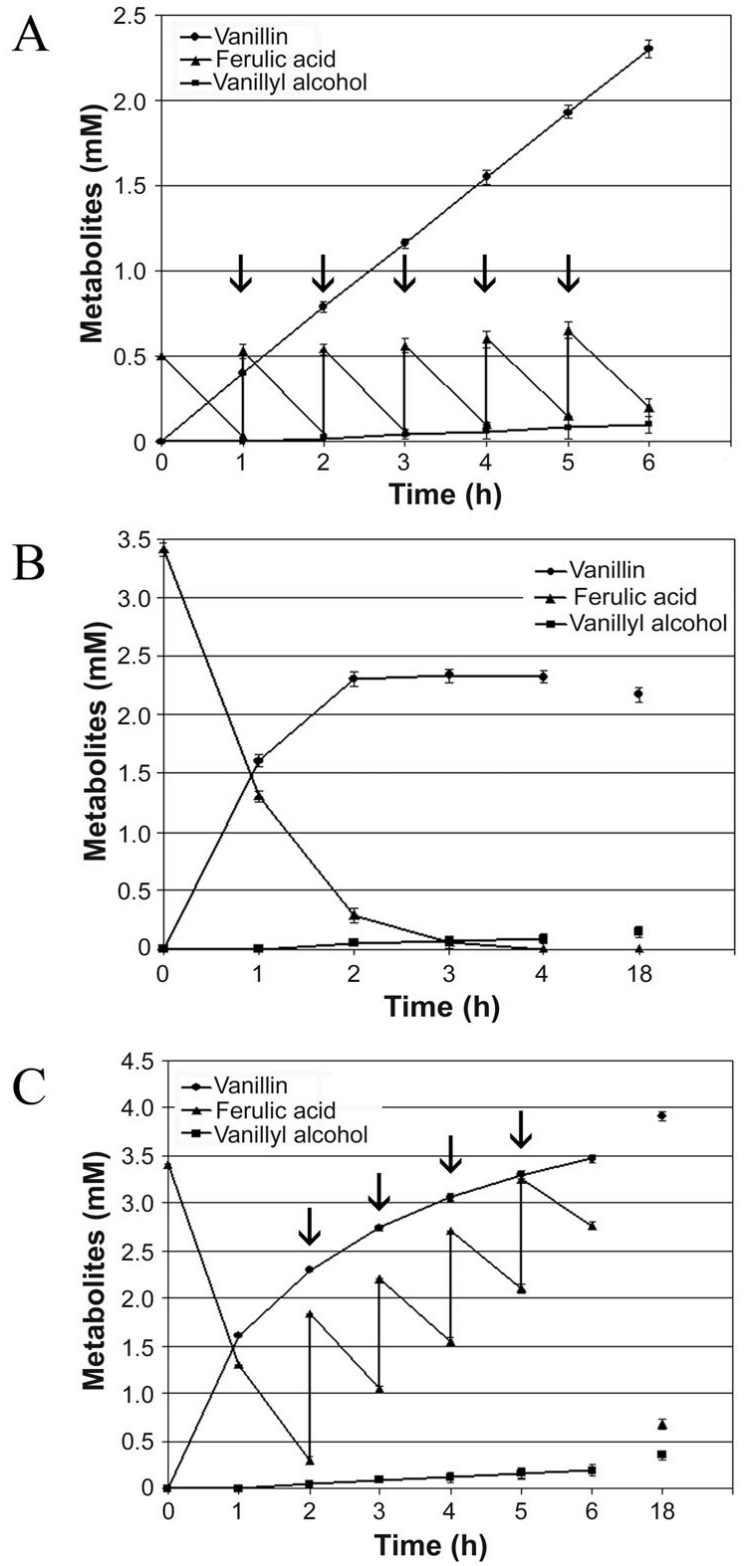
**Effect of ferulic acid concentration and mode of addition on the production of vanillin by resting cells [4.5 g(wet weight)/L] of JM109(pBB1) strain**. Experiments were carried out with (A and C) and without (B) pulse additions of the substrate and the initial concentration of the ferulic acid was 0.5 (A) or 3.3 mM (B and C), respectively. Arrows indicate the time for adding ferulic acid: 0.5 (A) or 1.1 (C) mmol/L, respectively.

Three-dimensional response surface curves were plotted to study the interaction between the two factors selected and to determine the optimum concentration of each for maximum vanillin yield and specific productivity (Figure 5A and 5B). The highest vanillin molar yield was achieved using 4.5 g (wet weight)/L of biomass and 0.5 mM ferulic acid (Table 1, experiment 2). Experimental yields obtained in three different experiments performed in these conditions were 75.0, 75.3 and 75.5% respectively, with a maximum vanillin concentration of 0.4 mM after 1 hour of incubation. Under these optimised conditions, with the aim of increasing vanillin production, ferulic acid was provided sequentially at 1-h intervals through pulses of 0.5 mmol/L for five times, for a total addition of ferulic acid of 3.1 mM. The vanillin produced after 6 hour of incubation was 2.33 mM (Figure 6A) with a yield of 75.2%. A slight amount of vanillyl alcohol (about 0.1 mM) was detected in the broth at the end of the incubation time.

The same methodology was also used to implement the specific productivity (Table 1). Surface of response for this variable in function of substrate and biomass concentration is presented in Figure 5B. The highest specific productivity was obtained with biomass concentration of 1 g (wet weight)/L and ferulic acid concentration of 2.8 mM, thus achieving a specific productivity of 6.92 μmol of vanillin produced/g of biomass × min (Table 1, experiment 4).

Considering both the molar yield and the specific productivity as responses, the model predicted that the best results (a molar yield of 69.5% and a specific productivity of 5.6 μmol of vanillin/g of biomass × min) could be obtained by employing 4.5 g (wet weight)/L of biomass and 3.3 mM ferulic acid (N° 4 shown in Table 2). The experimental results confirmed the mathematical model prediction; indeed, 2.33 mM vanillin with a molar yield of 70.6% and a specific productivity of 5.9 μmol of vanillin/g of biomass × min were obtained after 3 hours of incubation (Figure 6B). The concentration of vanillin did not increase by prolonging the incubation time, whereas vanillyl alcohol, that was less than 0.05 mM at this time, slightly increased upon incubation. A further attempt to increase vanillin production was made by employing the same experimental conditions [4.5 g (wet weight)/L biomass and 3.3 mM ferulic acid] by further addition of ferulic acid in 4 pulses of 1.1 mmol/L starting from the second hour of incubation for a total amount of added ferulic acid of 7.9 mM. After 6 hours of incubation, 3.5 mM vanillin was obtained while 2.7 mM ferulic acid was still present, and, after 18 hours, 4 mM vanillin and 0.7 mM ferulic acid were detected in the same reaction mixture (Figure 6C). The concentration of vanillyl alcohol remained low also at longer incubation times (about 0.3 mM).

**Table 2:** Predicted values of vanillin molar yield and specific productivity with various combinations of biomass and ferulic acid.

N°	Biomass (g/L)	Ferulic acid (mM)	Vanillin Molar yield (%)	Specific Productivity (μmol vanillin/g biomass × min)
1	4.88	2.75	70.87	5.22
2	4.73	1.62	73.36	3.89
3	1.40	3.20	56.40	6.99
4	4.50	3.30	69.50*	5.60*
5	4.87	2.80	70.72	5.27
6	4.77	1.79	73.02	4.15
7	1.44	3.22	56.57	6.97
8	1.44	3.22	56.57	6.97

*optimum conditions predicted by the model

**Figure 7:**
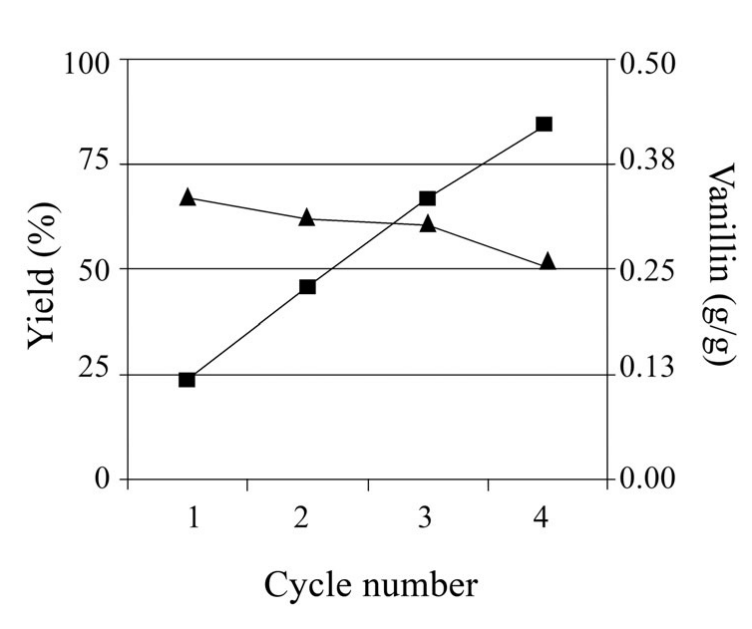
**Vanillin yield (filled triangle) and vanillin produced per gram of biomass (filled square), after successive cells reutilization processes**. Assays were performed with 4.5 g(wet weight)/L biomass and 5.2 mM ferulic acid.

As the biomass production was costly and time-consuming, the possibility of reusing the applied resting cells was also evaluated. Resting cells were reused four times (see Methods) achieving the vanillin production yields showed in Figure 7. Product yield remained over 50% until the fourth reuse, decreasing by only 17% in this interval. Figure 7 also shows that the reuse of the cells permitted to increase around three times the vanillin produced per gram of biomass.

## Discussion

The biotechnological production of vanillin is a topic of high interest, as demonstrated by the number of reviews published in the last years [1,2,5,6,19-24]. In the present study, the possibility of using recombinant *E. coli *strains, as an alternative to *Actinomyces *and Gram-negative strains usually employed, as biocatalysts for vanillin production was explored. It has been demonstrated [25] that the concerted expression of *fcs *and *ech *genes from *Pseudomonas *in *E. coli *confers the ability to convert ferulic acid to vanillin to the recombinant strain. We previously cloned the ferulic-catabolic genes from strain BF13 on a plasmid harbouring the broad-host-range RK2 minireplicon, so that they could be introduced and maintained both in *E. coli *and *Pseudomonas *cells [26]. Herein, the possibility of cloning these genes on a multicopy vector was explored and the effect of copy number amplification on vanillin production from ferulic acid was evaluated. Although the stability of the multi-copy ColE1 based plasmid (pFS) and the low-copy RK2 based plasmid (pBB1) in *E. coli *JM109 was comparable, JM109(pFS) cells were less efficient than JM109(pBB1) cells in bioconverting ferulic acid into vanillin (Figure 2). This result is consistent with observations published by Gasson et al. [27] on expression of feruloyl-CoA hydratase/lyase encoding-gene from *P. fluorescens *strain AN103 in *E. coli*. These authors showed that, when *ech *gene was placed under control of an IPTG-inducible promoter, higher levels of enzyme activity were achieved upon growing the recombinant strain in the absence of inducer with respect to induced cells. They postulated that increased protein expression occurred upon induction, but resulted in the production of incorrectly folded or inactive enzyme [27]. We suggest that the same effect is likely to occur with a 166-fold increase in copy number of ferulic-catabolic genes.

The production yield of vanillin could be increased using the low-copy plasmid containing cells collected from actively growing cultures (Figure 3) and performing the bioconversion experiments at 30°C (Figure 4). The strict dependence of ferulate catabolic gene expression and the physiological state of cells had already been observed in the *P. fluorescens *strain from which the genes had been isolated [18]. The indication that the highest yield of vanillin was obtained lowering the growth temperature from 37 to 30°C is in agreement with several reports on the sub-physiological temperature culturing (<37 degrees C) of *E. coli *cells as a strategy for increased recombinant protein yield [[28] and references cited therein]. A decrease in cultivation temperature affects replication, transcription and translation resulting in a decrease of the bacterial growth and in a reduction of cellular protein concentration, but, at the same time, reduces the harmful effects of the production of toxic stress proteins [28].

The response surface methodology (RSM) proved to be an effective way to further optimize the process. The highest vanillin molar yield (about 75%) was achieved at low ferulic acid concentrations (0.5 mM) using 4.5 g (wet weight)/L of biomass (Figure 5A). However, in these conditions, the maximum amount of vanillin that accumulated in the medium was low (0.4 mM) and this has a negative impact both on the product recovery and on the overall process economics. A 6.1-fold increase of vanillin concentration in the medium (2.33 mM) was obtained after 6 h incubation by pulse addition of ferulic acid, with no effect on the production yield (Figure 6A). The highest specific productivity (6.92 μmol of vanillin/g (wet weight) of biomass × min) was obtained with a low amount of biomass [1 g (wet weight)/L] and a substrate concentration of about 4 mM (Figure 5B). Nevertheless, the use of such a low amount of biomass would make the complete transformation of the substrate very slow, thus unacceptable for large scale productions, and could favour the transformation of vanillin into undesired products such as vanillyl alcohol or vanillic acid [14,16]. A favourable combination of both variables, corresponding to a substrate concentration of 3.3 mM and 4.5 g (wet weight) of biomass (Table 2), allowed to obtain a vanillin yield of 70.6% with a specific productivity of 0.354 mmoles of vanillin per gram of biomass per hour. Moreover, the same final vanillin concentration of 2.33 mM (about 355 mg/L) was produced in half of the time previously required, as it is evident from the comparison of Figure 6A and 6B. The effectiveness of the strategy based on pulse additions of ferulic acid was also effective under these optimized conditions, thus determining a further increase of the final vanillin production (Figure 6C) up to 3.5 mM, i.e. about 530 mg/L, after 6-hour incubation and 4 mM, i.e. about 600 mg/L, after 18-hour incubation. The decrease in vanillin bioconversion rate as incubation proceeded (Figure 6C) is probably due to the toxicity of vanillin for *E. coli *cells [16], which seems to be enhanced in the presence of high concentration (around 1.5 mM) of ferulic acid. The low residual concentration of ferulic acid after overnight incubation (18 hours) compared to the amount of vanillin produced, the low concentration of vanillyl alcohol evidenced and the absence of aromatic metabolites such as the CoA thioester may indicate vanillin transformation into non-aromatic metabolites at very long incubation times (Fig. 6C). However, the low tendency of *E. coli *JM109(pBB1) to reduce vanillin to the corresponding alcohol, which is a constitutive activity in *E. coli *strains [15] and often prevents vanillin accumulation in the broth [16], is a significant feature of this strain. As shown in Figure 6C, the product is stable after six hours of incubations.

Finally, the brief biotransformation time allowed the reuse of cells, which displayed a productivity higher than 50% after four cycles. A single biotransformation process required 11 h (4 h for cell growth and 7 h for both cell harvesting and the biotransformation process) and produced 0.13 g of vanillin per g of biomass. Biomass reuse for 4 cycles allowed to produce 0.42 g of vanillin in 32 h, thus enabling i) to reduce drastically the costs of production, and ii) to increase more than three-fold the vanillin produced per gram of cells. Considering that the yield of biomass, under the applied experimental conditions, was about 6 g per liter of culture, the final productivity of the system was 2.52 g of vanillin per liter of culture. This productivity is the highest reported up to now in literature for *E. coli *strains (1.1 g per liter of culture) after a comparable or longer incubation periods (48 h) [29]. Although the amount of vanillin produced is not comparable to actinomycetes productivity [7][8], two evidences, i.e. the easiness of cultivation of *E. coli *cells with respect to actinomycetes and the stability of vanillin produced by *E. coli *JM109(pBB1) with respect to the same product obtained by other Gram negative bacterial cells [10], clearly indicate the high potential of the biovanillin production system proposed in this study.

## Conclusion

The data collected suggest that the engineered strain *E. coli *JM109(pBB1) is a good catalyst for vanillin production from ferulic acid, as it efficiently bioconverts the target substrate into vanillin without accumulating undesirable vanillin reduction/oxidation products. The present work also represents the first study in which a large array of parameters are explored to optimize the bioconversion of ferulic acid to vanillin (physiological state of the cells, bioconversion temperature, biomass concentration, substrate concentration and mode of addition of ferulic acid, resting cells reusing strategy), leading to a productivity (2.52 g/L) which is the highest found in the literature for recombinant strains and the highest achieved so far applying such strains under resting cells conditions.

## Methods

### Chemicals

All chemicals and HPLC solvents were of the highest purity commercially available and were purchased from Fluka (Buchs, Switzerland) and Carlo Erba (Milan, Italy). Luria Bertani (LB) broth (Lennox L Broth) was from Acumedia (Baltimore, Maryland); Bacto-Agar was purchased from Difco (Detroit, Mich.).

### Microorganism cultivation

*E. coli *JM109 (*recA1 endA1 gyrA96 thi-1 hsdR17 *rk- mk+ *supE44 relA1 *λ^- ^*Δlac*-proAB F' *tra*D36 *pro*AB^+ ^*lacI*^*q *^*Z*ΔM15) was cultivated in LB broth at 37°C in Erlenmeyer flasks on an orbital shaker at 150 rpm. Solid media contained 1.5% (wt/vol) agar. Ampicillin and tetracycline were added at final concentrations of 100 and 25 μg/mL, respectively, for the growth of recombinant strains. Growth was monitored by measuring the turbidity of the cultures at 600 nm (OD_600_).

*E. coli *transformants were also grown in a 2-L fermenter (Applikon Italia, Genova, Italy) containing 1.1 L LB broth with 25 μg/mL tetracycline. The bioreactor was equipped with a stirrer containing two Rushton impellers and sterilizable probes to measure dissolved oxygen (pO_2_), pH and temperature. Cultivation was performed at 30°C with a mixing rate of 300 rpm and an aeration rate of 1.5 volume per volume per minute. An inoculum of 50 ml of an overnight preculture grown on LB with tetracycline was added to the fermenter in order to have an initial culture optical density of 0.2 at 600 nm.

### Cloning and DNA manipulations

Standard protocols were used for DNA cloning and transformation and for plasmid DNA purification [30]. Restriction endonuclease digestions and ligations with T4 DNA-ligase were done in accordance with the manufacturer's instructions (Invitrogen, Carlsbad, California, USA). The Wizard SV Gel and PCR Clean-up System (Promega, Madison, WI, USA) was used for the recovery of DNA fragments from agarose gels.

### Construction of recombinant plasmids

Plasmids used for metabolic engineering of *E. coli *were generated inserting a 5098-bp *Eco*RI-*Bam*HI fragment, which contained the first three genes of the ferulic catabolic operon (*ech*, *vdh *and *fcs*; GenBank accession number AJ536325) and the promoter region (P_fer_) from *P. fluorescens *BF13, into pGEM-3Zf(+), a high-copy pMB1 based vector, and pJB3Tc19, a low-copy RK2 based vector [31].

The 5098-bp catabolic cassette was generated in two steps. First, a 3938-bp *Sst*I fragment from p17Bam [32] was subcloned into pGEM-3Zf(+), generating a recombinant plasmid that contained P_fer_, *ech*, *vdh *and a truncated portion of *fcs*. The resulting plasmid was digested with *Sph*I and ligated with a 1160-bp *Sph*I fragment to reconstruct the entire coding region of *fcs*. The *Sph*I fragment was generated by enzymatic digestion of a PCR product encompassing the entire coding sequence of gene encoding feruloyl-CoA synthetase from strain BF13.

Inactivation of the vanillin dehydrogenase encoding gene was obtained by a frameshift mutation generated after digestion of plasmid molecules containing the 5098-bp catabolic cassette with *Bgl*II, fill-in reaction with "End Conversion Mix" (Novagen; Madison, WI) to produce blunt ends, and re-circularization, *in-vitro*, of the plasmid DNA with T4 DNA ligase. This frameshift mutation can be predicted to generate a truncated vanillin dehydrogenase that resulted to be unable to oxidize vanillin to vanillic acid.

Recombinant plasmids were introduced by transformation into *E. coli *JM109 cells and transformants were used in bioconversion experiments. The stability of the two plasmids in *E. coli *JM109 was high and comparable (more than 80% of cells retained the antibiotic resistance after 20 generations on LB medium without ampicillin).

### Preparation of template DNA for competitive PCR

Total intracellular DNA from plasmid-containing cells was extracted using the boiling method as described by Malorny et al. [33]. In brief, cells were grown to stationary phase in LB medium with ampicillin (100 μg/mL), collected by centrifugation, and resuspended in TE buffer (10 mM Tris-HCl, 0.1 mM EDTA [pH 8.0]) to a final density of about 2.5 × 10^10 ^cells/mL. The suspension was boiled for 10 min at 100°C, placed on ice for 5 min, and then centrifuged for 5 min at 15000 × g. After centrifugation, the supernatant containing DNA was transferred to a new microcentrifuge tube and 1-μl aliquot (2.5 × 10^7 ^cell equivalents) was used as the template for the PCR.

### Estimation of plasmid copy number

A 869-bp fragment of the gene encoding feruloyl hydratase/aldolase (*ech*) was used as the target DNA to determine plasmid copy number in JM109 recombinant strains. The target was amplified using *ech *specific primers: 5'-CGGGATCCGGCCGCTGATAGCTACGTTTG-3' and 5'-CGTTCTGCTCCCAGGTCAGCTC-3'. The competitor for the plasmid-encoded gene was constructed by deletion of an internal 138-bp *Nco*I fragment from the *ech *gene. Amplifications were performed in a total volume of 50 μl containing 250 μM dNTP, 1 μM of each primer, 1.5 mM MgCl_2_, 1× PCR buffer (20 mM Tris-HCl pH 8.0, 50 mM KCl), 1 μl of sample DNA (2.5 × 10^7 ^cells per reaction tube), 0.015 to 62.5 ng of competitor and 1.25 U *Taq *DNA polymerase (NewEngland BioLabs, Ipswich, MA). The following two-step PCR reactions were performed: one cycle at 94°C for 5 min, 26 cycles at 94°C for 30 s, 68°C for 90 s. To exclude the possibility of PCR inhibition, parallel positive control reactions using general primers targeting 16S rRNA genes (63F and 1389R; [34]) were conducted for each template. To ensure that we were working within the linear phase of each amplification reaction, aliquots of individual PCR reactions were removed at 3-cycle intervals, from 17 to 26 cycles, electrophoresed on 1% agarose gels, and stained with ethidium bromide. In the PCR in which the competitor and the target PCR products were at similar intensities, the target DNA and competitor DNA were assumed to be present at equimolar concentrations.

### Bioconversion experiments by resting cells of transformants

Cells were cultivated either in shaken flasks or in fermenter and, when the desired optical density was achieved, wet biomass was collected by centrifugation (6000 × *g *at 4°C), washed twice in M9 saline/phosphate buffer (4.2 mM Na_2_HPO_4_; 2.2 mM KH_2_PO_4_; 0.9 mM NaCl; 1.9 mM NH_4_Cl), and suspended in the bioconversion buffer (M9 buffer amended with 0.5 mg/L yeast extract [35]) in order to obtain a final concentration of biomass in a range from 1 to 8 g (wet weight)/L. In standard bioconversion process, a cell concentration of 4 g/L was employed. Biotransformations were performed in 100 mL flasks containing 10 mL of cell suspension supplemented with a sterile solution of ferulic acid in a range from 0.5 to 5 mM and incubated on a orbital shaker at 150 rpm. During bioconversion, 0.5 mL samples were withdrawn periodically and centrifuged at 15000 × g, and the supernatant analyzed to determine substrate and metabolite concentrations by high-pressure liquid chromatography. Reuse of resting cells was performed in bioconversion cycles of 6 h with 4.5 g/L biomass and 5.2 mM ferulic acid. Once the bioconversion finished, the cells were collected by centrifugation, washed and immediately used for the next bioconversion cycle. The reaction volume was adjusted to have the fixed amount of cells (4.5 g/L).

### Analytical methods

Substrates and metabolites occurring in culture supernatants were analyzed by liquid chromatography using a Varian ProStar high-pressure liquid chromatography (HPLC) apparatus equipped with a Nucleosil-100 C_18 _column, protected with a 1-cm guard cartridge (Phase Separations, Ltd.) and an UV-visible detector. Data acquisition and processing were controlled by the System Control Varian Star software (Varian, Palo Alto CA, USA). Metabolites were eluted with an isocratic method using, as mobile phase, an aqueous solution of methanol 35%, acetic acid 1%, at a flow rate of 1 mL/min. Sample detection was achieved at two wavelengths, 235 and 260 nm, and injection volumes were 20 μL. Compounds were identified comparing their retention time with those of authentic sample. They were eluted at the following retention time: vanillyl alcohol, 3.5 min; vanillic acid, 5.1 min; vanillin, 6.5 min; ferulic acid, 9.5 min. The identification of these compounds was also performed through GC-MS as reported by the Organic Chemistry Unit of Department of Agrobiolgy and Agrochemistry, University of Tuscia, Viterbo. For quantification, all intermediates were calibrated with external standards.

### Experimental design for optimization

The effect of biomass and substrate concentration on the vanillin production was determined by response surface methodology using a central composite design [36]. Statistical examination of results and generation of response surfaces were performed by the software package Modde 5.0 (Umetrics AB, Umea Sweden). Data were subjected to the analysis of variance (ANOVA) and fitted according to a second order polynomial model shown by equation:

Y = β_0 _+ Σβ_i_*X*_*i *_+ Σβ_ii_*X*_*i*_^2 ^+ Σβ_ij_*X*_*i*_*X*_*j*_

where Y is the predicted response variable, β_0 _is the intercept, β_i _and β_ii _linear coefficient and quadratic coefficients, respectively, β_ij _is the interaction coefficient and X_i _and X_j _are the coded forms of the input variables. To estimate the impact of single independent variables on the response, regardless of the presence of the other factors, main effects were calculated by the equation:

Y = β_0 _+ β_i_*X*_*i *_+ β_ii_*X*_*i*_^2^

The design was expanded to a circumscribed central composite design, being 12 the total number of experiments required for this experimental design, as shown in Table 1. The determination coefficient (*R*^2^) is the fraction of variation of the response explained by the model. The prediction coefficient (*Q*^2^) is the fraction of variation of the response that can be predicted by the model and provides the best summary of the fit of the model. Optimum reaction conditions predicted from such model, in terms of biomass and substrate concentration, (Table 2) were experimentally validated by HPLC quantitative analysis.

## Authors' contributions

PB and DD designed experiments, carried out analytical studies and wrote the manuscript. FF and MR contributed to experimental design and wrote the manuscript. All authors read and approved the final manuscript.
